# PWOs repress gene transcription by regulating chromatin structures in *Arabidopsis*

**DOI:** 10.1093/nar/gkae958

**Published:** 2024-11-11

**Authors:** Tingting Yang, Dingyue Wang, Lingxiao Luo, Xiaochang Yin, Zhihan Song, Minqi Yang, Yue Zhou

**Affiliations:** State Key Laboratory of Protein and Plant Gene Research, School of Advanced Agricultural Sciences, Peking-Tsinghua Center for Life Sciences, Peking University, No.5 Yiheyuan Road, Haidian District, Beijing 100871, China; State Key Laboratory of Protein and Plant Gene Research, School of Advanced Agricultural Sciences, Peking-Tsinghua Center for Life Sciences, Peking University, No.5 Yiheyuan Road, Haidian District, Beijing 100871, China; State Key Laboratory of Protein and Plant Gene Research, School of Advanced Agricultural Sciences, Peking-Tsinghua Center for Life Sciences, Peking University, No.5 Yiheyuan Road, Haidian District, Beijing 100871, China; State Key Laboratory of Protein and Plant Gene Research, School of Advanced Agricultural Sciences, Peking-Tsinghua Center for Life Sciences, Peking University, No.5 Yiheyuan Road, Haidian District, Beijing 100871, China; State Key Laboratory of Protein and Plant Gene Research, School of Advanced Agricultural Sciences, Peking-Tsinghua Center for Life Sciences, Peking University, No.5 Yiheyuan Road, Haidian District, Beijing 100871, China; State Key Laboratory of Protein and Plant Gene Research, School of Advanced Agricultural Sciences, Peking-Tsinghua Center for Life Sciences, Peking University, No.5 Yiheyuan Road, Haidian District, Beijing 100871, China; State Key Laboratory of Protein and Plant Gene Research, School of Advanced Agricultural Sciences, Peking-Tsinghua Center for Life Sciences, Peking University, No.5 Yiheyuan Road, Haidian District, Beijing 100871, China

## Abstract

PWWP-DOMAIN INTERACTOR OF POLYCOMBS (PWO) family proteins play a vital role in regulating plant development. However, the molecular mechanisms of how PWOs regulate chromatin structure is elusive. Our data show that the PWO1 binding sites are enriched with positive modifications but exclusive with H3K27me3. Moreover, PWO1 binds to the H3K27me3-enriched compartment domain (H3K27me3-CD) boundary regions, and functions to maintain the boundary strength. Meanwhile, we found that PWOs and Polycomb repressive complex 2 (PRC2) function parallelly in maintaining H3K27me3-CDs’ structure. Loss of either PWOs or PRC2 leads to H3K27me3-CD strength reduction, B to A compartment switching as well as the H3K27me3-CD relocating away from the nuclear periphery. Additionally, PWOs and lamin-like proteins collaborate to regulate multiple chromatin structures to repress gene transcription within H3K27me3-CDs. We conclude that PWOs maintain H3K27me3-CDs’ repressive state and regulate their spatial position in the nucleus.

## Graphical Abstract

**Graphical Abstract:**
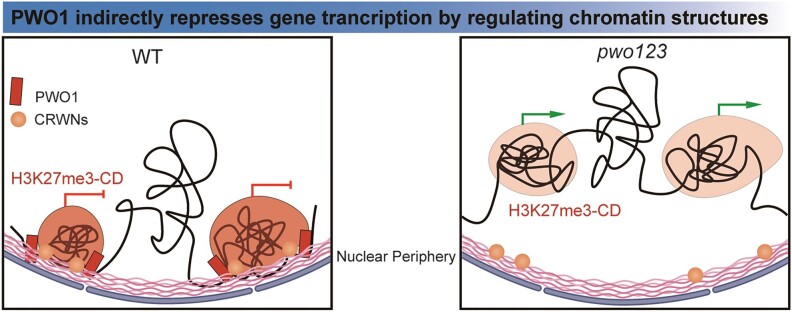

## Introduction

The plant specific PWWP-DOMAIN INTERACTOR OF POLYCOMBS (PWO) family consists of three highly redundant proteins, which play a vital role in developmental regulation; additionally, the *N*- and *C*-terminal domains of PWO1 have been reported to interact with Polycomb group (PcG) and lamin-like CROWDED NUCLEI 1 (CRWN1) protein, respectively (1,2). PcG proteins, first identified in *Drosophila*, are involved in cell fate determination, gene repression and chromatin 3D structure regulation (3–5). The repressive histone mark H3K27me3 is deposited by the Polycomb repressive complex 2 (PRC2). It is reported that PWOs may be involved in PRC2 recruitment (1). However, two new studies recently claimed that PWOs can also interact with UBIQUITIN SPECIFIC PROTEASE 5 (UBP5), which can remove H2Aub and antagonize PRC2-mediated repression (6,7). Therefore, the relationship between PWOs and PRC2/H3K27me3 is still not clear. Further research on the molecular mechanisms of how PWOs bind to chromatin and regulate gene transcription are required.

Chromatin 3D structure plays an important role in maintaining genome stability and regulating gene transcription (8–12). In mammalian cells, the formation of topologically-associated domains (TADs) is regulated by the CCCTC-binding factor (CTCF) and cohesin complex (9,13); however, the formation of TADs in *Drosophila* is correlated with chromatin states. Those TADs are regarded as the compartment domains (CDs), which are highly related to different histone modifications and can be divided into different types based on enriched modifications (14). Including the CTCF homolog in *Drosophila*, several architectural proteins are identified to bind to the domain boundary and regulate TAD structure (14–16). Although TADs or CDs have been identified in plants (5,17), the mechanisms underlying the regulation of chromatin 3D structure remain elusive. It has been reported that plant-specific Teosinte-branched 1/Cycloidea/Proliferating (TCP) transcription factors are enriched at domain (TAD or CD) boundaries in *Marchantia*, rice, maize and *Arabidopsis*; however, mutations in *TCPs* do not impair TAD or CD structure (18,19), indicating that the boundary regulation still needs to be studied. In addition, PRC1 and PRC2 are reported to regulate chromatin 3D structures (5). In mammals, H3K27me3-enriched regions form strong local interacting domains, as well as long-distance interactions (20,21). In plants, H3K27me3-enriched regions, such as the secondary metabolic gene clusters of terpenoids, are compacted as CDs (22). H3K27me3 is eliminated in its writer mutants (*clfswn or clf*), with concomitant loss of intra- and interdomain interaction intensity (12,23,24). High-throughput chromosome conformation Capture Hi-C in the H3K27me3 eraser mutant (*ref6*) further supports the vital roles of H3K27me3 in the establishment of local and long-range interactions (24); however, REF6 has not been reported to regulate H3K27me3-enriched compartment domains (H3K27me3-CDs) as a boundary-binding protein (25).

In mammals, hundreds of repressive chromatin domains, which connect closely with nuclear lamina, are clustered together to co-repress gene expression at nuclear periphery (26). Although there are no lamin proteins in *Arabidopsis*, three types of lamin-like proteins localized preferentially or exclusively at the inner nuclear membrane are found, that are CRWNs, KAKU4 and NUCLEAR ENVELOPE-ASSOCIATED PROTEINs. CRWNs have been reported to mediate repressive chromatin, especially centromere and pericentromeric regions, tethering near nuclear periphery (27). PcG and the histone modifications also have been confirmed to regulate H3K27me3-CDs and further influence the repressive state of such domains (5). However, the key regulator that bridges the H3K27me3-CDs to nuclear periphery has not been reported yet.

Here, we found that PWO1 directly binds to H3K27me3-CD boundary and bridges H3K27me3-CDs to nuclear periphery through physical interaction with CRWN proteins. PWOs, PRC2 and CRWNs cooperate to maintain the repressive chromatin state at nuclear periphery to repress gene transcription within H3K27me3-CDs.

## Materials and methods

### Plant materials and growth conditions

*Arabidopsis thaliana pwo1-1 pwo2-2*, *pwo2-2 pwo3-2* (1) were grown in a Percival growth chamber at 22°C under Long Day (LD) (16-h light/8-h dark) conditions. Seeds were incubated at 4°C in the dark for 2 days to stratify germination. All genotypes used in this study are in the Col-0 background. Crosses were performed with *pwo1-1 pwo2-2* and *pwo2-2 pwo3-2* to obtain the *pwo1-1 pwo2-2 pwo3-2* (*pwo123*) triple mutant. Homozygous mutants were identified by polymerase chain reaction (PCR)-based genotyping. To create the *pPWO1::PWO1-GFP/pwo1* transgenic plant, genomic fragment of PWO1 was cloned into the p407 entry vector. The transgenic plants expressed PWO1 protein fused with green fluorescent protein (GFP).

For RNA-sequencing (RNA-seq), chromatin immunoprecipitation sequencing (ChIP-seq), ATAC-sequencing (ATAC-seq), Micrococcal Nuclease-sequencing (MNase-seq) and Hi-C experiments, Col-0, *pwo1-1 pwo2-2* and *pwo1-1 pwo2-2 pwo3-2* seeds were sterilized in 75% ethanol and sown on Murashige and Skoog medium. Materials from 10-day-old seedlings were collected.

Transformation of *A. thaliana* was performed using the floral dip method and the *Agrobacterium tumefaciens* strain GV3101-pSOUP (28).

### Protoplast transfection

Protoplast transfection was performed following the Polyethylene glycol (PEG)-mediated protocol with some modifications (29). Plants were grown in Short Day (SD) (8-h light/16-h dark) conditions for 4 weeks, protoplasts were isolated from leaves by enzyme solution and kept at 4°C for 30 min. Protoplast pellets were then resuspended in MMG solution [(4 mM 4-Morpholineethanesulfonic acid hydrate (MES), 0.4 M mannitol and 15 mM MgCl_2_]. For *35S::CRWN1-FLAG*, *CRWN1* coding sequence was cloned into pXCSG-FLAG vector. For *i35S::PWO1-FLAG*, *PWO1* coding sequence was cloned into pER8-GW-FLAG (a β-estradiol-inducible 35S promoter vector, for oligonucleotide sequences, see Supplementary Table S1). For transfection assay, the construct *35S::CRWN1-FLAG* was transiently expressed in the protoplasts of wild type (WT) and *pwo12* mutant. Similarly, the construct *i35S::PWO1-FLAG* was transiently expressed in the protoplasts of WT and *crwn1* mutant. Subsequently, 40% PEG solution [0.2 M mannitol, 10 mM CaCl_2_ and PEG4000 (Sigma–Aldrich, USA)] was immediately added. The resulting solution comprising the protoplasts, plasmid DNA and PEG was gently mixed by inverting the tube, followed by incubation at room temperature for 12 min. The transfected protoplast mixture was washed thrice with 10 ml of chilled W5 solution (2 mM MES-KOH, 154 mM NaCl, 125 mM CaCl_2_ and 5 mM KCl), and incubated for 12–16 h at room temperature in weak light.

### RNA extraction and library preparation

Total RNA was extracted from 10-day-old seedlings under long-day conditions using an E.Z.N.A.® Plant RNA kit (Omega, R6827-01). RNA-seq libraries were prepared using a VAHTS Universal V8 RNA-seq Library Prep Kit for Illumina (Vazyme, NR605). Briefly, messenger RNA (mRNA) was purified using mRNA Capture Beads from 3 μg total RNA and fragmented in Frag/Prime buffer at 85°C for 6 min. Subsequently, double-stranded complementary DNA was synthesized, adapters were ligated and PCR amplification was performed for 13 cycles. Libraries were sequenced according to Novaseq 6000 guidelines (Illumina). Three biological replicates were performed for RNA-seq.

### ATAC-seq

For isolation of nuclei, 1 g fresh tissue was harvested and homogenized in ice-cold HBM buffer [25 mM Tris–HCl (pH 7.4), 0.44 M sucrose, 10 mM MgCl_2_, 0.1% Triton X-100, 2 mM spermine, 50× protease inhibitor cocktail and 1 mM Phenylmethylsulfonyl fluoride (PMSF)]. The sample was transferred through a 40 μm mesh and centrifuged for 2 min at 2000 × *g*, 4°C. After centrifugation, the nuclear pellet was resuspended and washed in HBB buffer [25 mM Tris–HCl (pH 7.4), 0.44 M sucrose, 10 mM MgCl_2_ and 0.1% Triton X-100]. Subsequently, 30 000 nuclei were counted and resuspended in 50 μl transposition reaction mix from the True-Prep™ DNA Library Prep Kit V2 for Illumina® (Vazyme, TD501). Reactions were incubated for 30 min at 37°C in a Thermal Cycler. DNA was then purified using VAHTS® DNA Clean Beads (Vazyme, N411). Two independent biological replicates were processed for next-generation sequencing library preparation. Libraries were prepared by PCR amplification for 13 cycles using two index (i5 and i7) Illumina barcodes (Vazyme, TD202). Libraries were size-selected using VAHTS® DNA Clean Beads. ATAC libraries were sequenced on an Illumina Hiseq-Xten PE150 by generating 2 × 150 bp paired-end reads.

### MNase-seq

MNase experiments were performed as previously described (30). Briefly, nuclei were extracted from 0.3 g material in Honda buffer [20 mM HEPES–KOH (pH 7.4), 0.44 M sucrose, 1.25% Ficoll, 2.5% Dextran T40, 10 mM MgCl_2_, 0.5% Triton X-100, 5 mM Dithiothreitol (DTT), 1 mM PMSF and protease inhibitor cocktail; Roche] and resuspended in MNase buffer [20 mM Tris–HCl (pH 8.0), 5 mM CaCl_2_ and 1 mM PMSF]. Approximately 500 000 nuclei were cleaved at 37°C for 10 min with 40 U micrococcal nuclease (Takara, 2910A). After stopping digestion, DNA was extracted using phenol–chloroform, followed by ethanol precipitation and treatment with RNase A at room temperature for 30 min. Libraries were then prepared using the NEBNext Ultra II DNA Library Prep Kit for Illumina (NEB, E7645S) and sequencing on an Illumina Nova Seq6000 by generating 2× 150 bp paired-end reads.

### *In situ* Hi-C and library preparation

*In situ* Hi-C was performed as previously described (30). Seedlings (10-day-old) were fixed in 1% formaldehyde in MC buffer [10 mM potassium phosphate (pH 7.0), 50 mM NaCl and 0.1 M sucrose] at room temperature, twice for 10 min each under a vacuum. After fixation, seedlings were subjected to a 5 min vacuum treatment with 0.1 M glycine. The fixed tissue was homogenized in liquid nitrogen, resuspended in nuclei isolation buffer [20 mM HEPES (pH 8.0), 250 mM sucrose, 1 mM MgCl_2_, 5 mM KCl, 40% (v/v) glycerol, 0.25% Triton X-100, 0.1 mM PMSF and 0.1% (v/v) ß-mercaptoethanol], and filtered through two layers of miracloth (Merck Millipore). Following resuspension in 0.5% sodium dodecyl sulphate (SDS), nuclei were denatured at 62°C for 5 min and then digested overnight with 50 U *Dpn*II at 37°C. The next day, digested DNA was blunt-ended using Klenow enzyme (Thermo Fisher Scientific), during which biotin-14-dCTP (Invitrogen) was incorporated. After ligation using T4 DNA ligase, DNA was isolated by phenol–chloroform extraction followed by ethanol precipitation and then sheared by sonication using a Bioruptor® Pico (Diagenode). Sheared DNA was size-selected (200–600 bp) using VAHTS® DNA Clean Beads (N411-01) and subsequently purified using Dynabeads™ MyOneTM Streptavidin C1 beads (Invitrogen). Following biotin enrichment, on-bead end-repair and adapter ligation were performed, the beads were washed and resuspended in 15 μl 10 mM Tris–HCl buffer (pH 8.0), and DNA was detached from the beads by incubation at 98°C for 10 min. Library molecules were amplified by 10 cycles of PCR, the products of which were purified using VAHTS® DNA Clean Beads (Vazyme, N411-01). Libraries were sequenced using an Illumina Hiseq-Xten PE150 platform by generating 2× 150 bp paired-end reads.

### Chromatin immunoprecipitation

Chromatin immunoprecipitation (ChIP) experiments were performed as previously described with some modifications (30). Briefly, 2.0 g 10-day-old seedlings grown under long-day conditions were harvested and fixed with 1% formaldehyde in MC buffer (described above). Seedlings were treated with 0.1 M glycine for 5 min in a vacuum to quench the reaction. The fixed tissue was homogenized in liquid nitrogen and resuspended in lysis buffer [50 mM HEPES (pH 7.5), 150 mM NaCl, 1 mM ethylenediaminetetraacetic acid, 0.1% deoxycholate, 0.1% SDS, 1% Triton X-100, 1 mM PMSF and 50× protease inhibitor cocktail]. Total chromatin was fragmented to sizes <500 bp by sonication using a Bioruptor® Pico (Diagenode) and subsequently immunoprecipitated with anti-GFP (Abcam, ab290) and anti-H3K27me3 (Millipore, 07–449) antibodies. Magna ChIP™ Protein A + G magnetic beads (Millipore, 16–663) were washed prior to being used to capture GFP- or H3K27me3-associated DNA. After decrosslinking, DNA was isolated by phenol–chloroform extraction followed by ethanol precipitation.

For ChIP-seq samples, two biologically independent DNA libraries were constructed using the Ovation® Ultralow System V2 and sequenced using a Hiseq-Xten PE150 platform.

For ChIP-qPCR, three biological replicates were performed. Fold enrichment of each examined DNA fragment was normalized to the input using the ΔCt method. *ACT2* was used as the negative control. PCR primers are described in Supplementary Table S1.

### 3D FISH

The FISH experiment was carried out as described previously with some modifications (23,31). Nuclei were extracted from 10-day-old *Arabidopsis* seedlings. Nick translation of bacterial artificial chromosome (BAC) JAtY73L13 (Chr. 5: 19422491–19495524) was used to prepare the digoxigenin-11-dUTP (Roche, 11175033910)-labeled probes. Primary and fluorescently labeled secondary antibodies were applied to detect digoxigenin-11-dUTP: mouse anti-digoxin antibody (Sigma–Aldrich, D-8156) (1:500) and goat anti-mouse antibody coupled to Alexa Fluor 488 (Invitrogen, A11017) (1:150), respectively. SlowFade™ Diamond Antifade Mountant with DAPI (Invitrogen, S36964) was used to label nuclei. A Zeiss LSM 900 Airyscan 2 system and a 63× oil objective was used to detect fluorescent signals and take FISH images. Images were processed using the ZEN Microscopy Software. Nuclear periphery association was signified by all signal spots in one nucleus overlapping with the nuclear periphery.

### RNA-seq data analysis

We first applied fastp to filter the raw sequencing data and then mapped the reads with gaps to the reference genome (TAIR10, https://www.arabidopsis.org) using Hisat2 (32). After filtering, indexing and converting the format using SAMtools (33), we employed StringTie (34) for transcript quantitation and then extracted the read counts using the Python script prepDE.py provided by StringTie (34). The R package DESeq2 (35) was subsequently used to analyze differentially expression (*q* < 0.05 and fold change > 0, unless otherwise noted), which takes a matrix of read counts mapped to genes in reference annotation as input (Araport11, https://www.arabidopsis.org). The reproducibility of sequencing data was verified using principal component analysis generated from the top 1000 differentially expressed genes (DEGs) and visualized in ggplot2 (http://ggplot2.tidyverse.org). Gene ontology was analyzed and plotted using clusterProfiler (36).

### ChIP-seq, ATAC-seq and MNase-seq data analysis

Fastp (37) was used to filter out low-quality raw reads, after which clean reads were mapped back to the *Arabidopsis* reference genome (TAIR10, https://www.arabidopsis.org) using Bowtie2 (38). Mapped reads were indexed, cleaned by removal of reads mapped to plasmids and the format was converted using SAMtools (33). Duplications were subsequently removed using PICARD (http://broadinstitute.github.io/picard/) and significant peaks (*q*< 0.01) were calculated using macs2 (39). Protein binding sites are narrow peaks that were calculated using default parameters; H3K27me3-modified regions are broad peaks that were calculated using the parameter –nomodel –bw 300 –broad. BEDtools (40) was applied to extract the read coverage followed by DESeq2 to calculate differentially modified genes, which were then visualized in ggplot2. Annotation and visualization of the peak binding features were performed using R package ChIPpeakAnno (41). Venn diagrams were visualized using R package VennDiagram (42) and statistically assessed using a hypergeometric test. We visualized the modifications in the WashU Epigenome Browser (43). The meta gene plots were produced using Seqplots (44). Further, for MNase-seq analysis, the DANPOS2 (45) dpos function was applied to sample the read count to the same depth, normalize by fold change, and obtain smoothed wig format files containing averaged nucleosome occupancy values at each base pair across the entire genome.

### Modification enrichment analysis

Analysis was performed as previously described (30). Briefly, the public histone modifications were obtained from Plant Chromatin State Database (46) and listed in the Supplementary Table S2. We calculated the coverage of each selected modifications on target and random selected regions by Bedmap with -wmean (47). The significant enrichment or depletion of each modification were evaluated by pairwise Mann–Whitney *U*-tests and Bonferroni–Holm method for multiple comparisons (48). Other next-generation sequencing data used in this study were taken from the NCBI database (https://www.ncbi.nlm.nih.gov/), the details of which can be found in Supplementary Table S2.

### Hi-C data analysis

Raw sequencing data were filtered using fastp and the reads were mapped back to the reference genome (TAIR10, https://www.arabidopsis.org) using Bowtie2 (38). The valid contacts were filtered and identified using HiC-Pro (49) with default parameters. The contact matrixes were visualized using HiCdataR (50) with iterative normalized contact matrixes. Subsequently, HiCExplorer (51) accepted the HiC-Pro output and then hicNormalize with—smallest was applied to normalize the sequencing depth and hicCorrectMatrix was used to correct the matrix with KR (Knight and Ruiz) (52) balancing methods [Sun *et al.* (53) was referred to for Hi-C data analysis methods]. The interaction decay exponents were calculated using hicPlotDistVsCounts and visualized in R using the ggplot2 package (53). After calculating the TAD separation score using hicFindTAD with parameters –minDepth 3000 –maxDepth 30 000 –step 1000 –thresholdComparisons 0.05 –delta 0.01 –correctForMultipleTesting fdr, we annotated the H3K27me3-CDs with strong boundaries and interaction intensities that overlapped with chromatin states enriched in H3K27me3 marks (chromatin state 5 regions) (54). The heatmaps for protein binding, and H3K27me3 levels in the H3K27me3-CDs regions were computed and visualized using deepTools (55). The intradomain contacts was visualized by GENOVA (56) and quantfied by hicInterIntra (51). The TAD-separation scores for the boundaries were visualized in SeqPlots (44).

## Results

### PWO1 binding sites are mutually exclusive with H3K27me3

**Figure 1.**
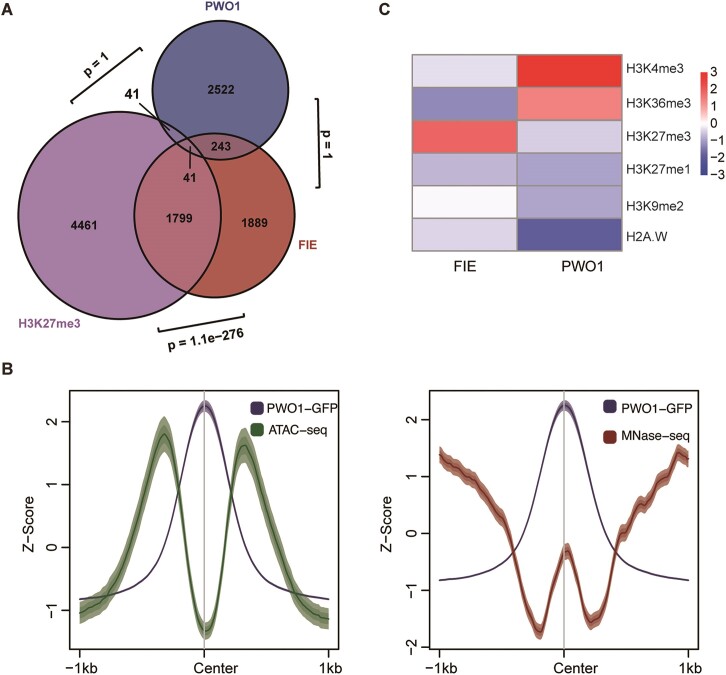
PWO1 is mutually exclusive with H3K27me3 and associated with positive modifications. (**A**) Venn diagrams showed overlap between PWO1 target genes and H3K27me3-marked genes or FIE target genes. Using the number of protein-coding genes annotated in the genome (27655) as the sample size, we applied a hypergeometric test to calculate the *P*-value. (**B**) Meta plots showed the signals of PWO1-GFP and ATAC-seq (left panel) or MNase-seq (right panel). The PWO1-GFP, ATAC-seq or MNase-seq signals are indicated by blue, green or red lines, respectively. The fields represent the standard error and 95% confidence interval. (**C**) Heatmap indicated the relative enrichment or depletion of each histone modification in Col-0 among the FIE- (left panel) or PWO1-occupied (right panel) regions. Randomly selected regions were compared. The Wilcoxon rank sum test was applied to calculate the significance of enrichment or depletion. Published data were obtained from the Plant Chromatin State Database and are listed in Supplementary Table S2.

PWOs are essential for *Arabidopsis* developmental regulation, which is indicated by the seedling lethal phenotype of the *pwo123* mutant (Supplementary Figure S1). In contrast, *pwo1* single mutant does not show obvious phenotype (1). Therefore, it is meaningful to clarify the molecular mechanisms by which PWOs bind to chromatin and regulate gene expression. We performed transcriptomics analysis with *pwo1* and *pwo123* mutants. Our results showed that although the number of misregulated genes in *pwo1* mutants are less than that in *pwo123*, the DEGs are significantly overlapped between two mutants (Supplementary Figures S2 and S3), which indicates PWO1/2/3 have similar function. To study the genome-wide distribution of PWOs, we performed PWO1-GFP ChIP-seq experiments with *pPWO1::PWO1-GFP/pwo1* plants (Supplementary Figure S4). The results showed that PWO1 binds to chromosome arm regions instead of pericentromere regions (Supplementary Figure S5). In addition, the meta plot and the corresponding heatmap showed that PWO1 prefers to bind to the transcription start site region, and close to +1 nucleosome (Supplementary Figure S6). It has been reported that PWO proteins possess the capacity to recruit PcG proteins for the repression of target gene transcription (1,2). However, according to our genome-wide ChIP-seq data, the distribution of PWO1 is surprisingly separated from that of H3K27me3-marked genes, PRC2 core components FERTILIZATION INDEPENDENT ENDOSPERM (FIE) and CURLY LEAF (CLF) target genes (Figure 1A, and Supplementary Figure S7). Through combined analysis, we found that the cluster of binding sites for PWO1 is located at the nucleosome occupied regions (Figure 1B), whereas a typical DNA-binding protein displays an opposite curve shape, reflecting preference for binding to the regions depleted of nucleosomes (Supplementary Figure S8) (57). This observation implied the potential binding of PWOs is close to the nucleosomes, rather than binding to the chromatin open regions. Therefore, we examined the enrichment of histone modifications at PWO1 binding sites to find some clues. The results showed that PWO1 binding sites are mostly enriched in positive modifications and depleted in repressive histone modifications (Figure 1C). In contrast, the binding sites of FIE, which is the core component of PRC2, are mostly enriched with H3K27me3 (Figure 1C). Taken together, our results suggest that the PWO1 prefers to bind to the regions enriched with positive modifications but exclusive with H3K27me3.

### PWO1 binds to the H3K27me3-CD boundary and maintains boundary strength

**Figure 2.**
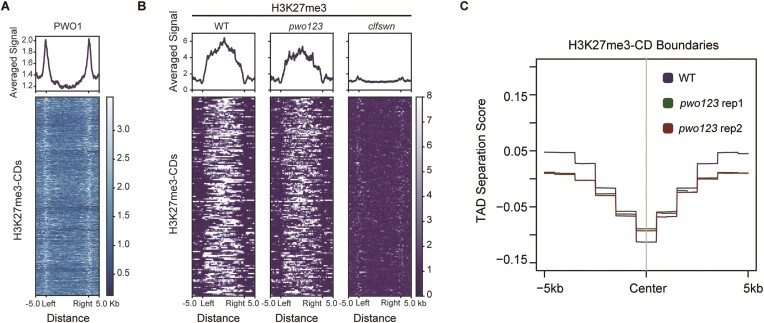
PWO1 binds to H3K27me3-CD boundaries. (**A**) Meta plot and the corresponding heatmap indicated enrichment of the average PWO1 ChIP-seq signal among H3K27me3-CDs. (**B**) Meta plots and the corresponding heatmaps showed H3K27me3 levels among H3K27me3-CDs. From left to right: WT, the *pwo123* and *clfswn* mutants. (**C**) Meta plot showed the aggregated H3K27me3-CD separation score at H3K27me3-CD boundaries. The Wilcoxon rank sum test was applied to calculate the *P*-value of TAD separation score at the center of the boundaries. *P* = 1.077e−13 for WT and *pwo123* replicate 1; *P* = 1.918e−12 for WT and *pwo123* replicate 2; *P* = 0.5084 for *pwo123* replicate 1 and *pwo123* replicate 2.

PcG proteins are important for the regulation of chromatin 3D structure (12,24). As the PcG antagonists (6,7), we investigated whether PWOs are involved in the regulation of chromatin 3D structure using Hi-C in WT and the *pwo123* mutant. The reproducibility was further confirmed by scatter plot analysis (Supplementary Figure S9). In order to investigate the regulatory mechanism of PWO1 binding and H3K27me3, we annotated H3K27me3-regulated chromatin domain structure (hereafter as H3K27me3-CD) by chromatin state (54). Combined with PWO1-GFP ChIP-seq data, we found that the all H3K27me3-CDs are enriched with PWO1 bindings at boundaries (Figure 2A, and Supplementary Table S3). There are only 4.0% (113/2803, Supplementary Table S4) of PWO1 binding sites within H3K27me3-CDs, which consists with the exclusive relationship between PWO1 binding sites and H3K27me3 (Figure 1A). Compared with the elimination of H3K27me3 in *clfswn* mutant, removal of PWOs does not impair H3K27me3 enrichment within H3K27me3-CD, since H3K27me3 levels within H3K27me3-CD are just slightly lower in the *pwo123* mutant (Figure 2B). Considering the boundary binding ability of PWO1, we further investigated whether the boundary strength is changed in the *pwo123* mutants. We calculated the TAD separation score, which is applied to identify CD boundaries (53). The lower value of TAD separation score represents the higher boundary strength. The meta plot clearly showed a higher TAD separation score in the *pwo123* mutant, illustrating the intensity of boundary strength is significantly decreased (Figure 2C). These results indicate that PWO1 prefers to bind to the boundary rather than the inside regions of H3K27me3-CD, and functions to maintain the boundary strength of H3K27me3-CDs.

### PWO1 and PRC2 parallelly contribute to regulate multiple chromatin structures and maintain H3K27me3-CDs spatial position

**Figure 3.**
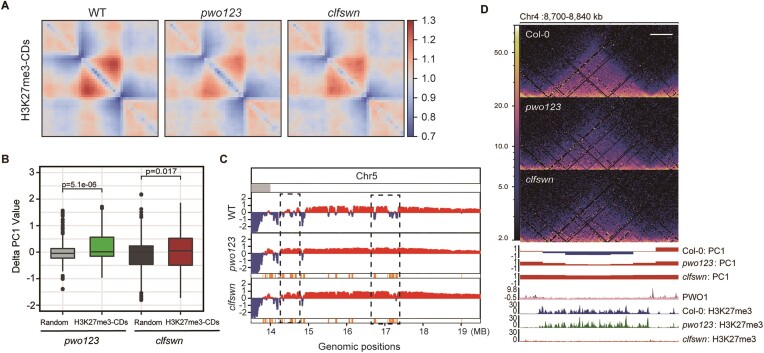
PWOs regulate multiple chromatin structures. (**A**) ATA plots indicated the raw interactions of WT, the *pwo123* and *clfswn* mutant among the H3K27me3-CDs with PWO1 binding at the boundary. (**B**) Box plot showed the delta (*pwo123* - WT) PC1 values of H3K27me3-CD regions in comparison with randomly selected regions. The Wilcoxon rank sum test was applied to calculate the *P*-value. (**C**) The tracks showed PC1 values generated for each 20 kb genomic segment from Hi-C data on the right arm of chromosome 5 (Chr5). Negative PC1 values indicate B compartments (blue), and positive PC1 values indicate A compartments (red). The tracks below the PC1 values represent B to A switch regions (orange). Dashed boxes indicate the switched regions. (**D**) The contact matrix, compartment, PWO1 occupancy and H3K27me3 modification as shown from top to bottom. Scale bar indicates 20 kb in length as shown by a white line at the top right of the matrixes. The negative PC1 values indicate B compartments, and positive PC1 values indicate A compartments. ChIP-seq signals are shown in BPM (Bins Per Million mapped reads). ChIP-seq of PWO1-GFP and the input signal are shown on same track in dark and light purple, respectively.

Boundary regulation is important for the TAD or CD structure regulation (9,10). Since the intensity of boundary strength is reduced in the *pwo123* mutant, we investigated the strength of H3K27me3-CD in the *pwo123* mutant. The aggregate TAD analysis (ATA) result showed that interaction intensity is decreased within H3K27me3-CD in the *pwo123* as well as *clfswn* mutant in comparison with WT plants (Figure 3A). In contrast, the strength of other CD is almost unchanged in the *pwo123* mutant compared with WT (Supplementary Figure S10), which indicates that PWOs specifically regulate H3K27me3-CDs. Additionally, we discovered that the repressive state of H3K27me3-CD is disrupted in the *pwo123* and *clfswn* mutants, since the first principal component (PC1) value within H3K27me3-CD is significantly increased while random regions do not show a similar change (Figure 3B). PC1 values are used to determine the A/B compartment status of a given region (30). Increase of PC1 value in the *pwo123* and *clfswn* mutants implies a general B to A compartment switching (Figure 3C and D). In summary, our results suggest that both PWOs and PRC2 are involved in maintaining the strength of H3K27me3-CD and B compartment.

In order to rule out the possibility that H3K27me3-CDs changes are caused by severe phenotype of *pwo123*, we performed Hi-C and H3K27me3 ChIP-seq in *pwo12* mutant. Our results showed that, similar to *pwo123* mutant, H3K27me3 level within H3K27me3-CDs is slightly lower than WT plants (Supplementary Figure S11A). The interaction intensity is decreased within H3K27me3-CD in the *pwo12* mutant in comparison with WT plants (Supplementary Figure S11B and E). Meanwhile, PC1 values within H3K27me3-CDs are significantly increased in the *pwo12* mutants, which led to B to A compartment switching (Supplementary Figure S11C–E). Moreover, we used published H3K27me3 ChIP-seq and Hi-C data of *clf* mutant to analyze whether chromatin structures are affected in *clf* mutant. The meta plot and heatmap showed that H3K27me3 level within H3K27me3-CDs is decreased compared with WT plants (Supplementary Figure S11A). However, the interaction intensity and PC1 values within H3K27me3-CDs remains the same as WT plants (Supplementary Figure S11B–E). These results indicated that only reduction of H3K27me3 does not affect chromatin structures (*clf* mutant), but elimination of H3K27me3 or mutation in *PWO1/2* or *PWO1/2/3* significantly affects chromatin structure (*clfswn*, *pwo12* or *pwo123* mutants).

**Figure 4.**
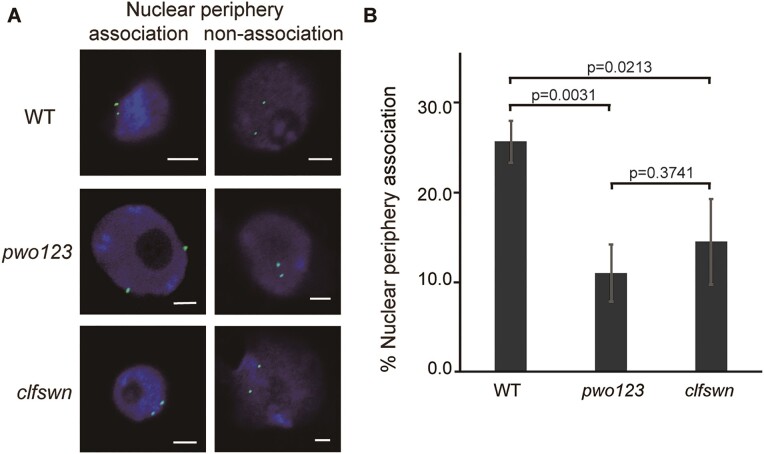
PWOs and H3K27me3 function in concert to regulate H3K27me3 spatial position. (**A**) and (**B**) 3D DNA FISH analysis of the nuclear periphery association of a typical H3K27me3-CD. Representative images (**A**) and percentage of nuclear periphery association (**B**) for bacterial artificial chromosome (BAC) JAtY73L13 (Chr. 5: 19422491–19495524, Supplementary Figure S12) are shown. Scale bar, 2 μm. Three biological replicates were performed to calculate the nuclear periphery association. *P*-value is calculated by ANOVA.

In *Arabidopsis*, the repressive state of H3K27me3-CD is strongly maintained by the nuclear periphery association (27,31). We wondered whether PWOs connect H3K27me3-CDs to the periphery of the nucleus. We used GFP-labeled BAC to detect one of the H3K27me3-CDs’ spatial position (Supplementary Figure S12). Although 3D-FISH results showed GFP signals were associated with or without nuclear periphery in WT or the *pwo123* mutant (Figure 4A), the percentage of nuclear periphery association is significantly decreased in the *pwo123* mutant (Figure 4B). Meanwhile, we found that the percentage of nuclear periphery association is also significantly reduced in PRC2 (*clfswn*) mutant (Figure 4B). Our data indicated that H3K27me3 is almost unchanged in the *pwo123* mutant or eliminated in the *clfswn* mutant (Figure 2B), but the percentage of nuclear periphery association is similar in the two mutants, which suggested that PRC2/H3K27me3 and PWOs parallelly contribute to maintain the H3K27me3-CD special position and the repressive chromatin state.

### PWOs and CRWNs collaborate to regulate chromatin structures and indirectly repress gene transcription

**Figure 5.**
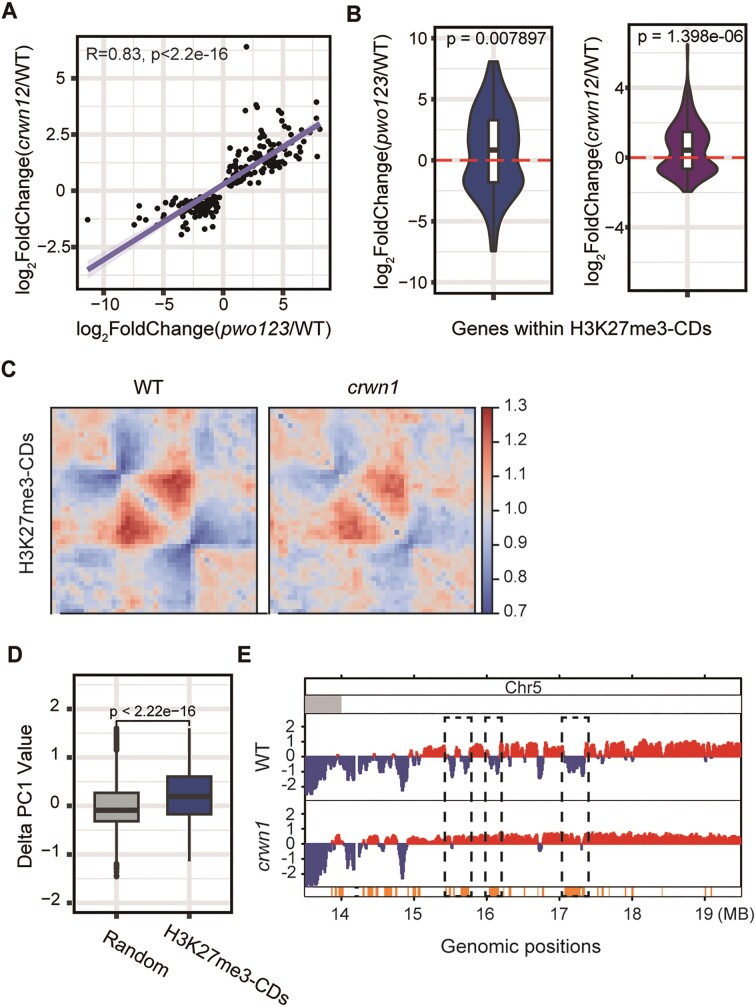
PWOs and CRWN1 collaborate to regulate chromatin structures and indirectly repress gene transcription. (**A**) Scatter plot indicated the positive correlation of coregulated genes within H3K27me3-CDs between the *pwo123* and *crwn12* mutants. (**B**) Violin plots indicated the log_2_ fold change in RNA-seq level at the genes that are coregulated by PWOs or CRWNs within H3K27me3-CDs in the *pwo123* or *crwn12* (2) mutants. The one sample *t-*test was applied to calculate the *P*-value. (**C**) ATA plots indicated the raw interactions of WT and the *crwn1* mutant among the H3K27me3-CDs. Hi-C data for the *crwn1* mutant and corresponding WT was obtained from the database and are listed in Supplementary Table S2. (**D**) Box plot showed the delta (*crwn1* - WT) PC1 values of H3K27me3-CD regions in comparison with randomly selected regions. The Wilcoxon rank sum test was applied to calculate the *P*-value. (**E**) The tracks showed PC1 values that generated for each 20 kb genomic segment from Hi-C data on the right arm of Chr5. Negative PC1 values indicate B compartments, and positive PC1 values indicate A compartments. The tracks below the PC1 values represent B to A switch regions. Dashed boxes indicate the switched regions.

CRWN proteins localize at the nuclear periphery and function to link chromatin repressed regions and nuclear periphery (2,27). Since PWOs are reported to interact with CRWN proteins (2), we then investigated whether PWOs and CRWNs work together to regulate gene transcription. The RNA-seq results showed that all the DEGs as well as the DEGs within H3K27me3-CDs are significant overlapped in the *pwo123* and *crwn12* mutants (Supplementary Figure S13). Moreover, the overlapped genes within H3K27me3-CDs showed positive correlation (Figure 5A). Transcription levels of these genes tend to upregulate in the *pwo123* and *crwn12* mutants (Figure 5B), which suggests that PWOs and CRWNs collaborate to repress gene transcription. Since PWO1 prefers not to bind inside of H3K27me3-CDs (Figure 2A), these genes are not the direct targets of PWO1. When we checked the binding of CRWN1 (58), the results showed that binding sites of CRWN1 distribute within H3K27me3-CDs (Supplementary Figure S14A). PWO1 and CRWN1 are always adjacent to each other (Supplementary Figure S15A). Our ChIP-PCR results showed that the binding ability of PWO1 is independent on CRWN1, since the binding of PWO1 was not affected in *crwn1* mutant (Supplementary Figure S15B and D). However, CRWN1 binding is significantly reduced in *pwo12* mutant (Supplementary Figure S15C and D). It has been reported that CRWN1 is involved in regulation of plant nuclear lamina-associated domains (PLADs) (58). We found PWO1 also binds to the boundary of PLADs (Supplementary Figure S14B). The H3K27me3-CDs and PLADs are significantly overlapped (Supplementary Figure S14C). In addition, our results showed that the chromatin structure changes are similar in the *pwo123* and *crwn1* mutants. Firstly, the strength of the H3K27me3-CDs whose boundaries are bound by PWO1 is decreased in the *crwn1* mutant (Figure 5C); secondly, the PC1 value is also significantly increased within H3K27me3-CD in the *crwn1* mutant (Figure 5D). The increase of PC1 value indicates the B to A compartment switching, which explained why the continual A compartment regions could be observed in the *crwn1* mutant (Figure 5E). In summary, these results demonstrate that the interaction between PWOs and CRWNs contribute to repress gene transcription by maintaining the silent chromatin state.

## Discussion

A confusing phenomenon is that PWOs interact with both H3K27me3 writer (CLF) (1) and H2AK121ub erasers (UBP5) (6,7). H3K27me3 and H2AK121ub are both repressive modifications deposited by PcG complexes. The biological relevance that PWOs interact with two functionally opposite proteins is unclear, which is interesting and worth exploring. It has been reported that PWOs may recruit PcG to target genes and repress gene transcription. However, our ChIP-seq results showed that PWO1 binding sites are mutually exclusive with H3K27me3/PRC2. The significant exclusive between PWOs and H3K27me3/PRC2 demonstrates that function of PWOs is not related with PcG recruitment. Previous study showed that PcG proteins can form PcG body by liquid–liquid phase separation (LLPS) in mice and human cell lines (59,60). Regions of DNA within PcG bodies that PcG-bound contact frequently with each other. Further evidence implicated that genes were derepressed when PcG body formation was perturbed, for example, deletion or mutation of the SAM domain of PH in *Drosophila* (61). The existing data showed that PWO1 interact with PRC2, and recruit CLF to form nuclear speckles through LLPS in N. *benthamiana* leaves (1). Although PWO1 binds to the boundary of H3K27me3-CD and PRC2/H3K27me3 distributes inside the H3K27me3-CD, the interaction between PWOs and PRC2 may contribute to compact H3K27me3-CD from boundary to the inside region and form the PWOs-PRC2 droplets in the nucleus. The relation of PWOs and PRC2 is independent, since the H3K27me3 is almost unchanged in *pwo123* mutant. In addition, we provided evidence to show that PWOs interact with CRWNs to drive H3K27me3-CDs to nuclear periphery in *Arabidopsis*. PWOs and CRWNs collaborate to regulated multiple chromatin structures and repress gene transcription within H3K27me3-CDs. In animals, compartment B is mostly located at the nuclear lamina, while compartment A is primarily located in the nuclear interior (26). Our data suggests PWOs are the bridge proteins and function to regulate spatial position of H3K27me3-CDs in the nucleus.

Boundary is significant for chromatin 3D structure regulation. In mammals, CTCF binds to the boundary to maintain TAD structures. In *Drosophila*; however, not only are the various architectural proteins such as dCTCF and BEAF-32 (14) involved in the TAD and boundary regulation, but also the *cis*-elements (62,63). However, the boundary regulation of chromatin 3D structures remains unknown in plants. In *Arabidopsis*, the homolog of canonical insulator CFCF is absent and there are no defined architectural proteins as in *Drosophila*. Research on chromatin 3D structure in plants has made some progress in recent years. In this research, we found that PWOs bind to the boundary of H3K27me3-CD. The PWO1 binding sites and H3K27me3 are exclusive with each other, which explains why PWO1 prefers not to bind within H3K27me3-CD. Loss of PWOs influences the multiple chromatin structures. First, depletion of PWOs weakens the boundary strength of H3K27me3-CD which further leads to the reduction of the H3K27me3-CD strength; secondly, the repressive B compartment state is disrupted in the *pwo123* mutant; thirdly, PWOs cooperate with CRWNs to co-repress gene transcription inside H3K27me3-CDs. In this study, our working model highlights that PWOs act as a bridge to closely connect H3K27me3-CDs and the periphery regions of the nucleus by physical interacting with CRWN1 to maintain the repressive chromatin state. Our results showed that CRWN1 binding is reduced in *pwo12* mutant, which explained the lower percentage of nuclear periphery association in the *pwo123* mutant. Meanwhile, PWO1 binding is not affected in *crwn1* mutant. CRWNs are reported to form the homo- and/or hetero- oligomers for construction of the meshwork structure (64). Additionally, according to the phenotypic effects of combining mutations, four CRWNs were shown to contribute to some essential processes such as specifying nuclear size (65). We think CRWNs have similar functions. Therefore, we cannot exclude the possibility that PWO1 binding would be affected when mutated more *CRWNs*. Moreover, how PWOs maintain boundary strength is unclear. The interaction between PWOs and UBP5 may account for maintaining the strength of boundary and H3K27me3-CD. Both our and published data showed that PWOs and UBP5 binding sites are associated with active histone modifications rather than H3K27me3 (6,7). We provide two possible roles for the interaction between PWOs and UBP5. Firstly, it has been recently reported that UBP5-mediated H2A deubiquitination prevents deposition of H3K27me3. We speculate that UBP5 may target to the H3K27me3-CD boundary and prevent the spreading of H3K27me3. The interaction between UBP5 and PWOs restricts H3K27me3 within H3K27me3-CDs and to maintain the H3K27me3-CD’s boundary. In mammalian cells, CTCF proteins restrict the spreading of H3K4me3 into the repressive domains and maintain the TAD structure simultaneously (66). Another possibility is that UBP5 functions to maintain the H3K27me3 level within H3K27me3-CDs. In addition to UBP5, there are other two H2A deubiquitinase in *Arabidopsis*, UBP12 and UBP13. It has been reported that the removal of H2Aub1 by UBP12/13 prevents loss of H3K27me3 in some genomic regions (67). UBP5 may also function through the similar mechanism as UBP12/13 at some certain regions such as H3K27me3-CD regions.

## Supplementary Material

gkae958_Supplemental_Files

## Data Availability

All sequencing data generated in this study have been deposited into the Genome Sequence Archive (GSA) in National Genomics Data Center (NGDC) under the accession number CRA016242 that are publicly accessible at https://ngdc.cncb.ac.cn/gsa. Our previously generated data have been deposited into the European Nucleotide Archive (ENA) under the accession number PRJEB52473 (Hi-C in WT and *clfswn*).
