# Supplementary material for: PWOs repress gene transcription by regulating chromatin structures in Arabidopsis
Source: Nucleic Acids Res. 2024 Nov 11;52(21):12918–29. doi: 10.1093/nar/gkae958 (PMC11602166; doi:10.1093/nar/gkae958)
Supplement: gkae958_Supplemental_Files [file gkae958_supplemental_files.zip › Supplementary information-clean.docx]

**Supplementary information**

**
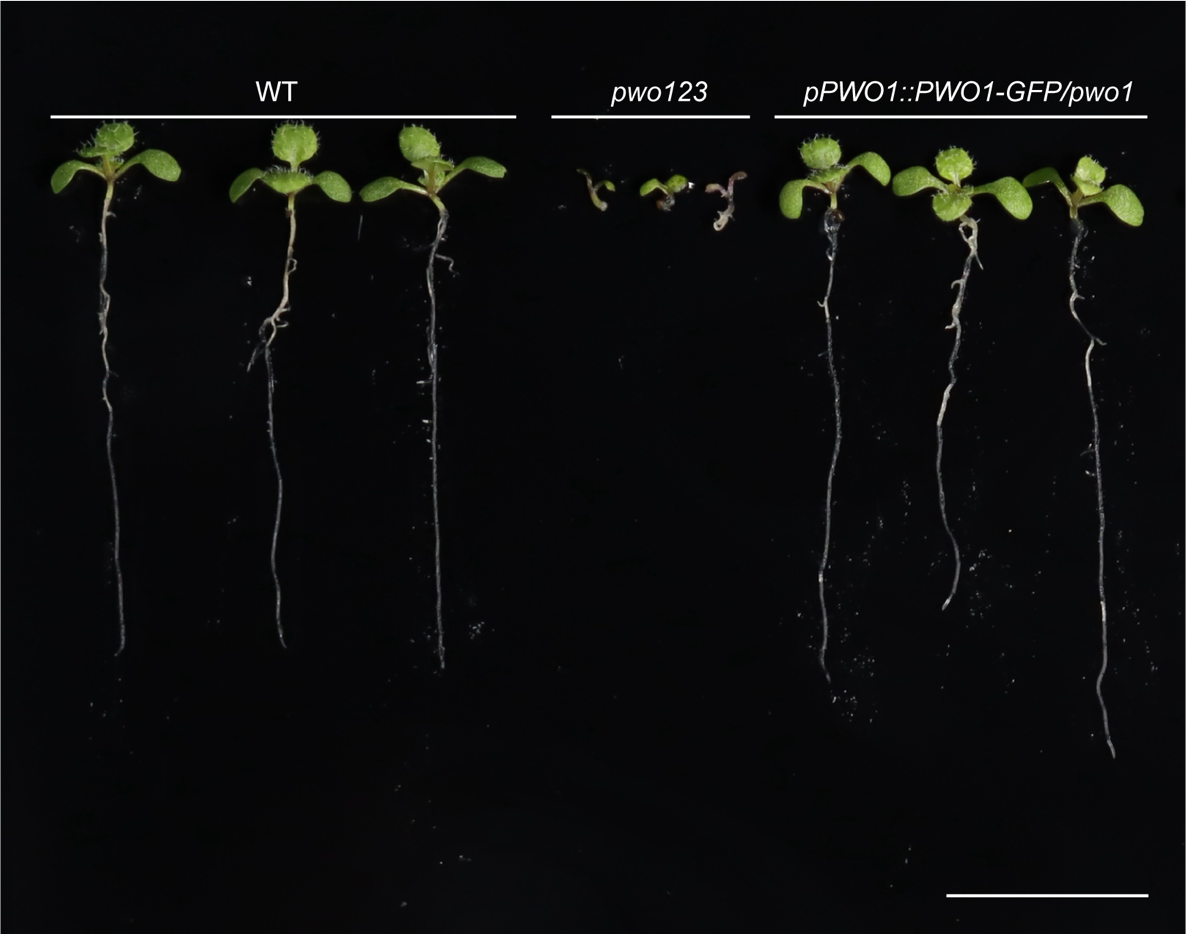
**

**Fig. S1 Phenotype of WT, the *pwo123* mutant, and transgenic plants.** Developmental analysis of WT, the *pwo123* mutant and *pPWO1::PWO1-GFP/pwo1* were performed at 10 days after germination. Scale bar: 1 cm.


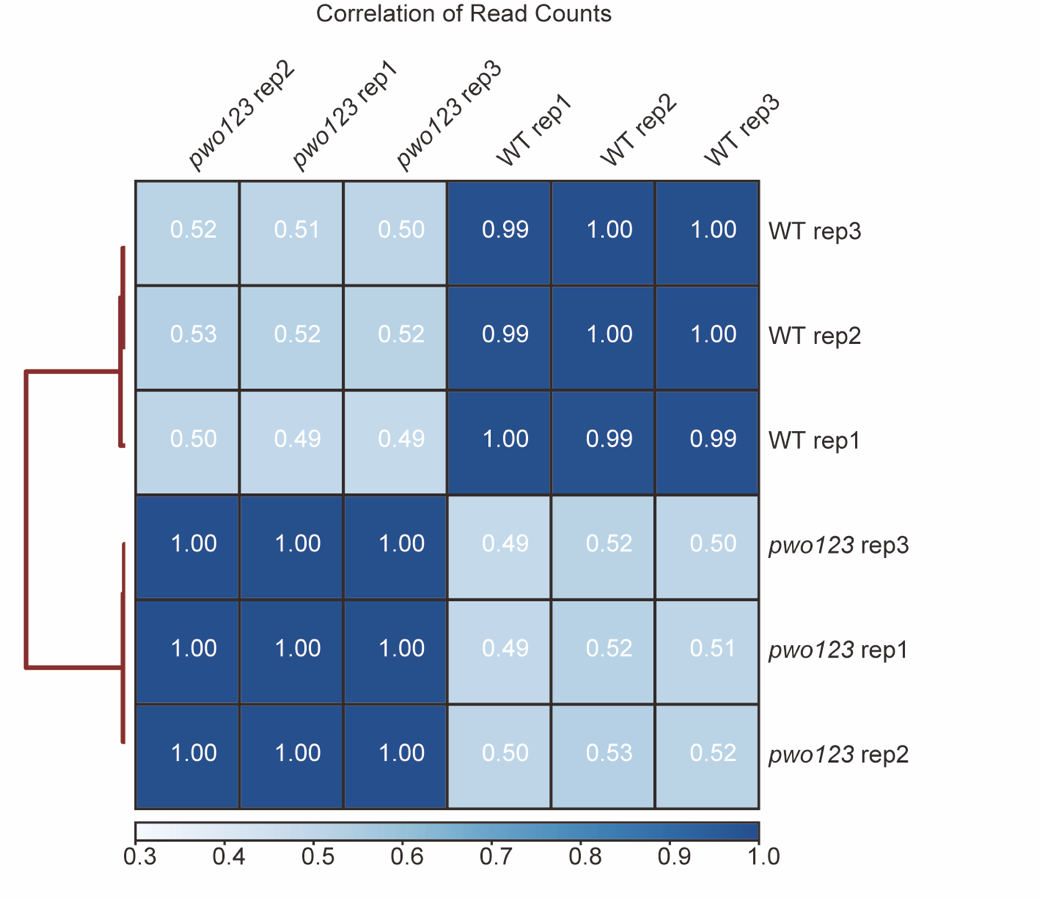


**Fig. S2 Repeatability test of RNA-seq.** Correlation of RNA-seq read counts of WT and the *pwo123* mutant was shown. Repeatability test of RNA-seq was applied by Pearson correlation.

**
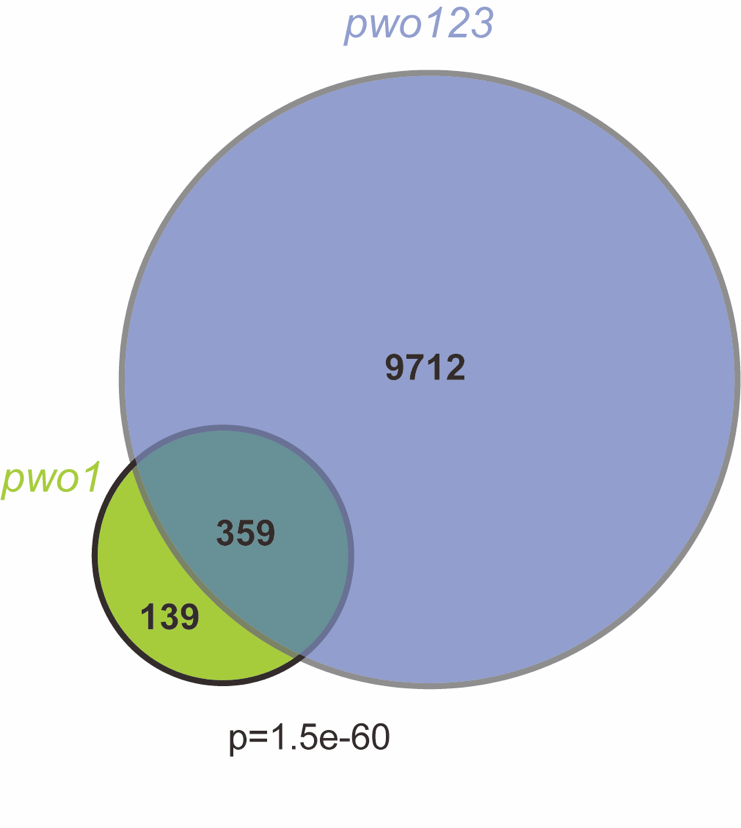
**

**Fig. S3 Differentially expressed genes between *pwo1* and *pwo123*.** Venn diagrams showed the overlapped differentially expressed genes between *pwo1* and *pwo123* (log_2_foldchange > 1; q < 0.05). We applied a hypergeometric test to calculate the p value.

**
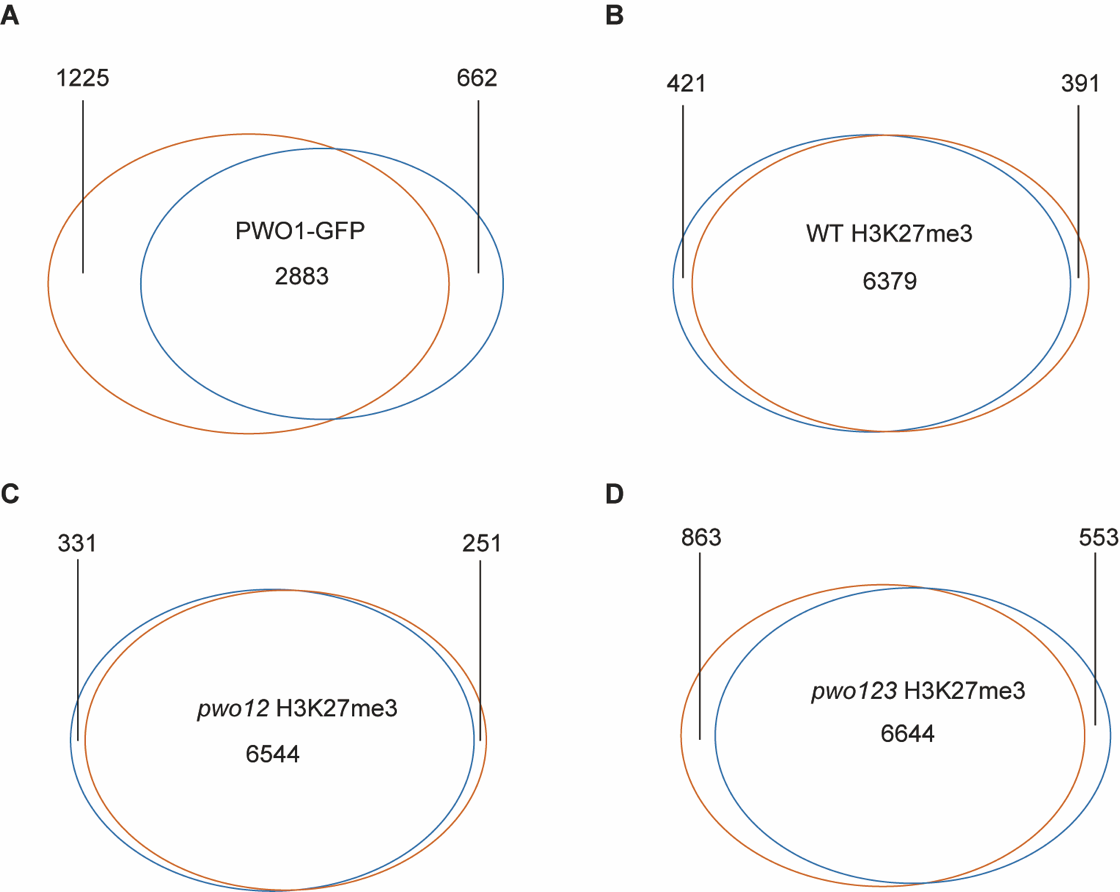
**

**Fig. S4 The results of ChIP-seq are repeatable among replicates.** The peaks from each replicate are overlapped, which indicated high-quality of ChIP-seq results for PWO1-GFP (A), H3K27me3 in WT (B), *pwo12* (C) and *pwo123* mutants (D).

**
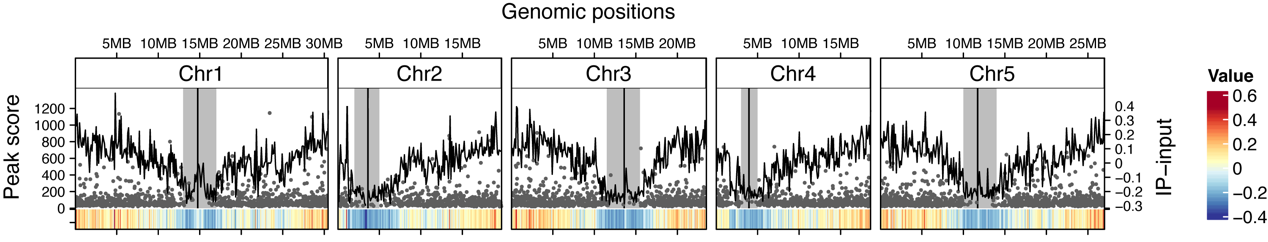
**

**Fig. S5 Genome-wide view of PWO1 binding and the peak score.** The PWO1-GFP signal (IP - input) is indicated by black lines and the heatmap at bottom track. A gradient change from blue to red in the heatmap indicated the PWO1-GFP signal (IP - input) strength from weak to strong. Gray dots indicated the significant peaks called by macs2. Pericentromere regions and the centromere position of each chromosome are indicated by light gray and black strips, respectively.

**
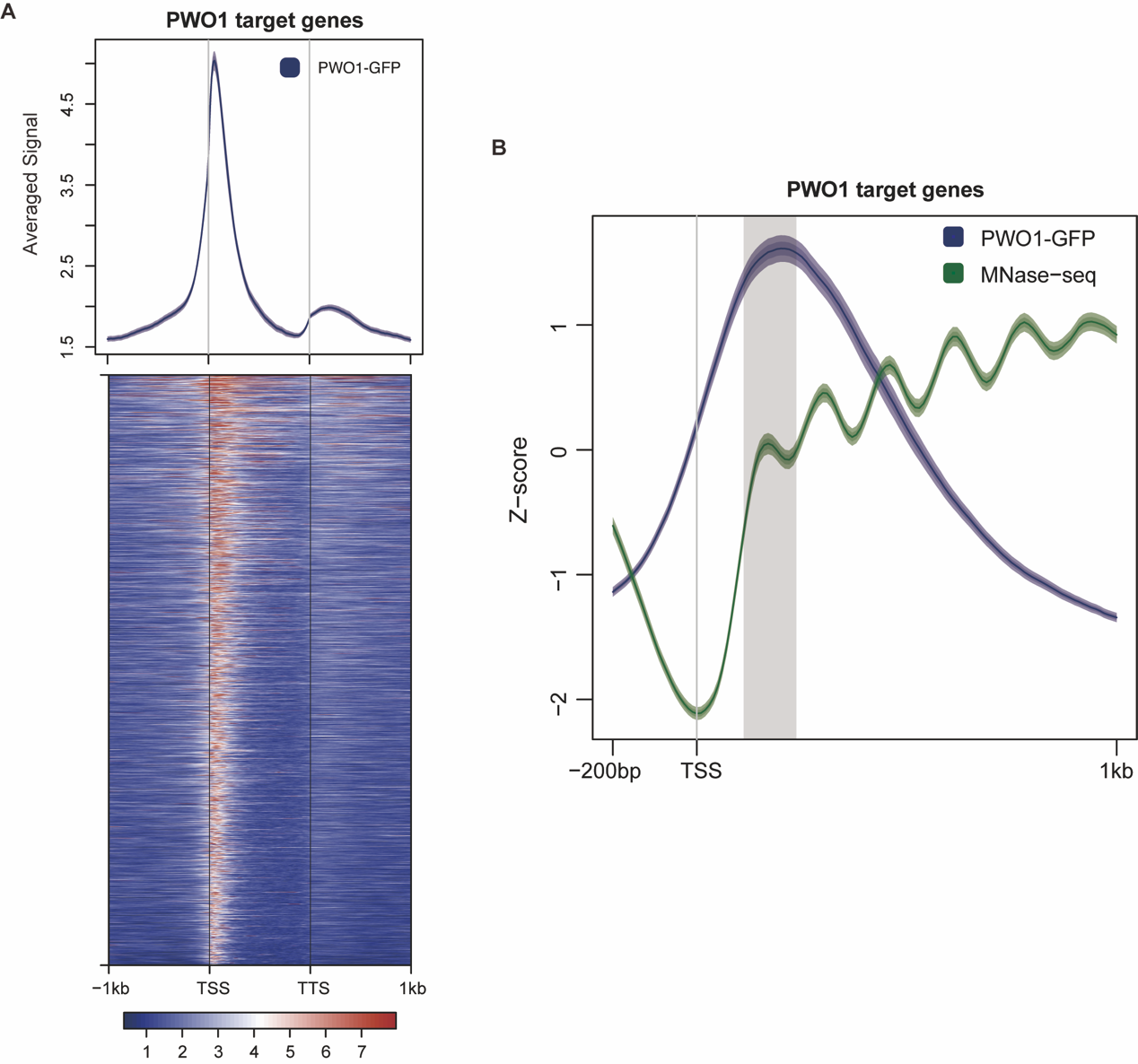
**

**Fig. S6 PWO1 binding profile at target gene regions**. **A** The meta plot and corresponding heatmap indicated that PWO1 preferred to target to TSS flanking region of target genes. **B** Meta plot showed that the Z-score normalized of PWO1-GFP (blue) and MNase-seq (green) signals at transcription start site (TSS) of PWO1 target genes. The peak of PWO1 binding is close to the +1 nucleosome.


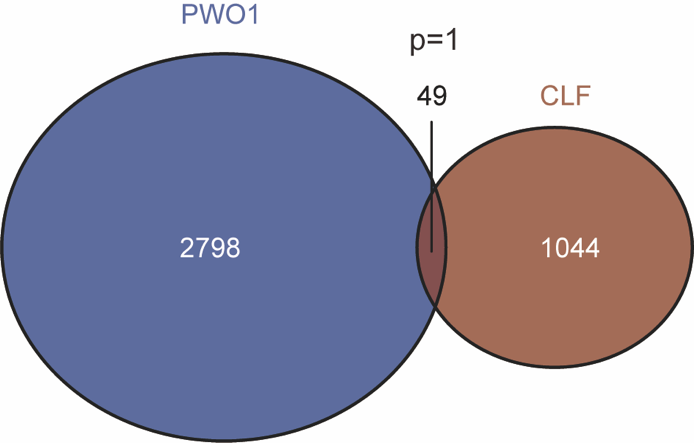


**Fig. S7 PWO1 and CLF target genes are exclusive with each other.** Venn diagram showed PWO1 target genes are independent of CLF target genes. We applied a hypergeometric test to calculate the p value.


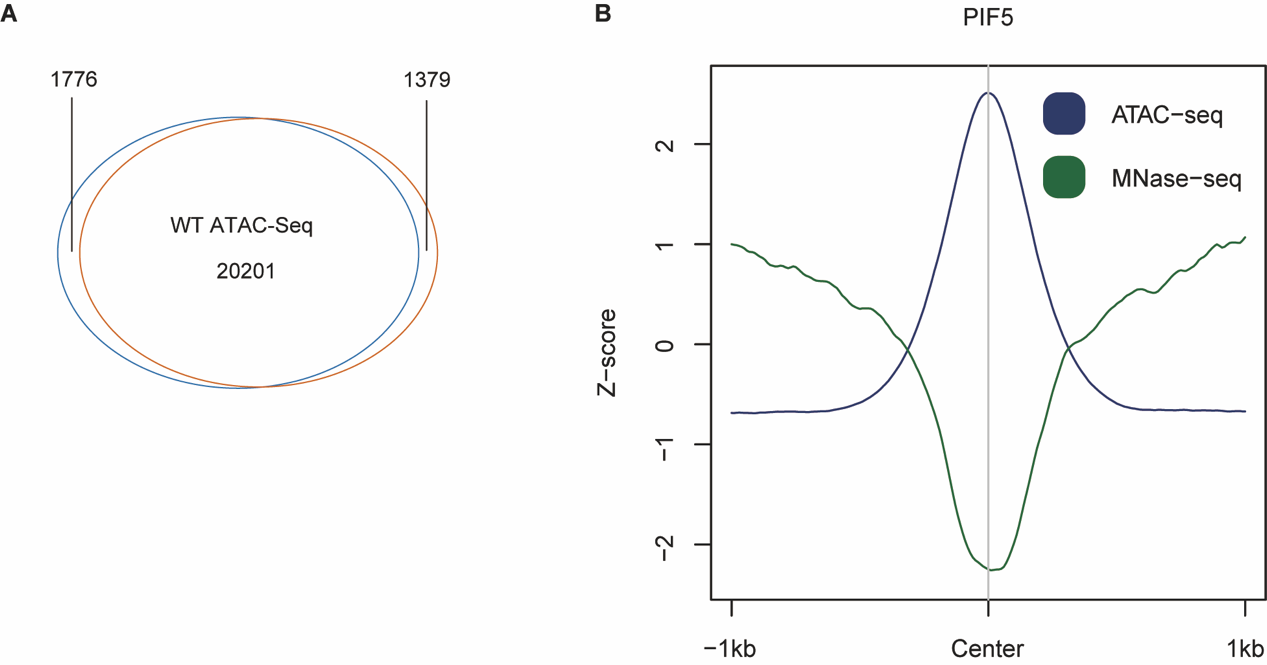


**Fig. S8 ATAC-seq and MNase-seq results indicate PIF5 binds to the nucleosome depletion region. A** The quality of ATAC-seq was verified by the presence of sufficient peaks and repeatability between replicates. **B** PIF5 prefers to bind nucleosome depletion region. Blue and green lines indicate the Z-score signals of ATAC-seq and MNase-seq, respectively.


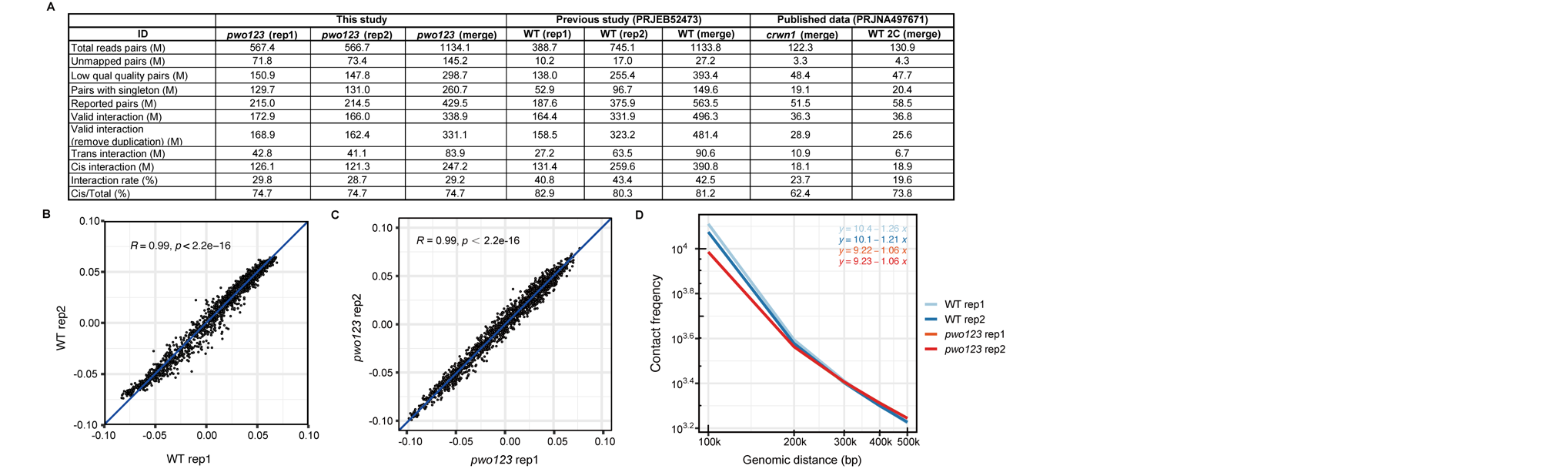


**Fig. S9 Hi-C results for the *pwo123* mutant. A** Statistical analysis of valid interactions in the *pwo123* mutant. **B and C** Scatter plots of WT (B) and the *pwo123* mutant (C) showed the reproducibility of two biological replicates. Each point represents the PC1 value of compartment at 20 kb resolution. **D** IDEs showed the difference in contact frequency between WT and the *pwo123* mutant in chromosome arm regions.

**
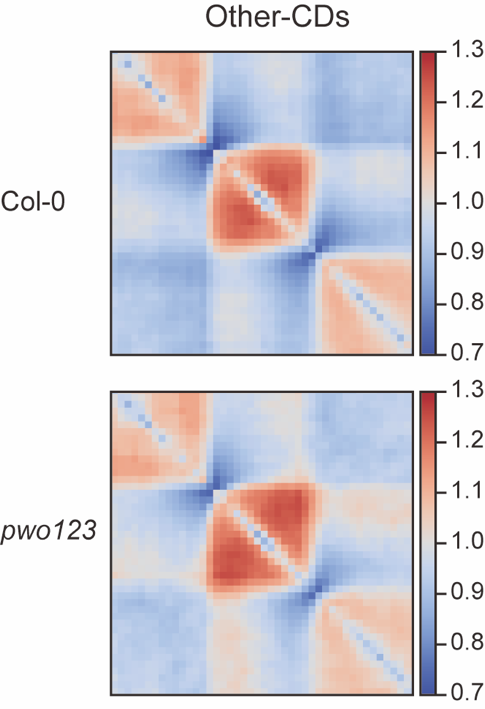
**

**Fig. S10 PWOs are not involved in regulating other-CDs.** ATA plots indicated the raw interactions of WT and the *pwo123* mutant among the other-CDs.


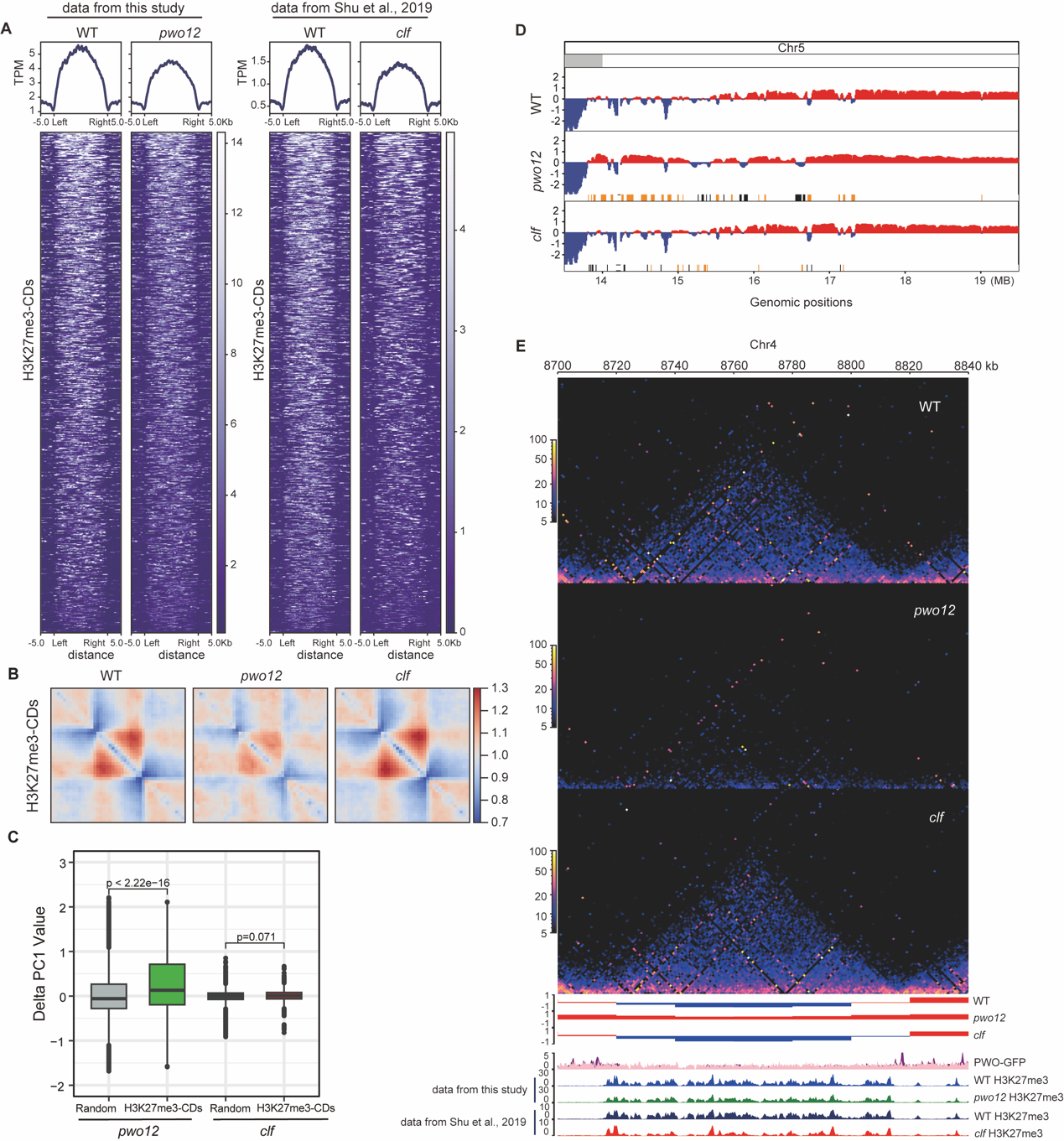


**Fig. S11 Chromatin structures in *pwo12* and *clf* mutants. A** H3K27me3 levels decreased slightly in *pwo12* and *clf* mutants compared with WT plants. **B** ATA plots indicated the raw interactions of WT, the *pwo12* and *clf* mutants within the H3K27me3-CDs. **C** Box plot showed the delta (mutant - WT) PC1 values of H3K27me3-CD regions in comparison with randomly selected regions. The Wilcoxon rank sum test was applied to calculate the p value. **D** The tracks showed PC1 values generated for each 20 kb genomic segment from Hi-C data on the right arm of chromosome 5 (Chr5). Negative PC1 values indicate B compartments (blue), and positive PC1 values indicate A compartments (red). The tracks below the PC1 values represent B to A switch regions (orange) and A to B switch regions (black). **E** The contact matrix, compartment, PWO1 occupancy, and H3K27me3 modification were shown from top to bottom. The red and blue blocks under the matrix represent A and B compartments, respectively. ChIP-seq signals were shown in BPM (Bins Per Million mapped reads). ChIP-seq of PWO1-GFP and the input signal were shown on same track in dark and light purple, respectively.

**
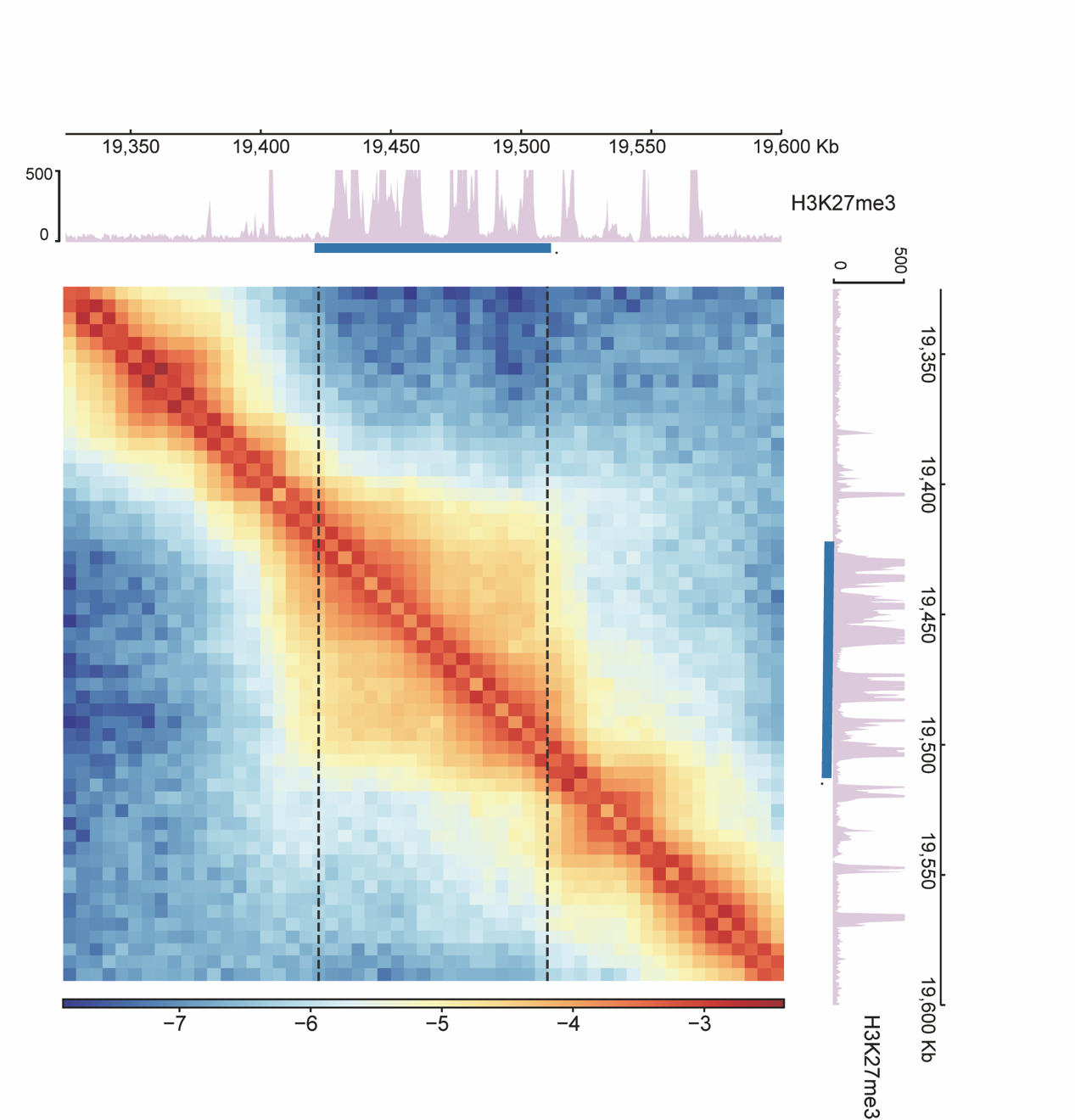
**

**Fig. S12 The position of BAC probe used for FISH.** Heatmap showed the H3K27me3-CD that selected to detect the nuclear periphery association by FISH. Blue blocks on the top and right indicate BAC position (JAtY73L13; Chr. 5: 19422491-19495524). ChIP-seq of H3K27me3 signal are shown in RPKM.


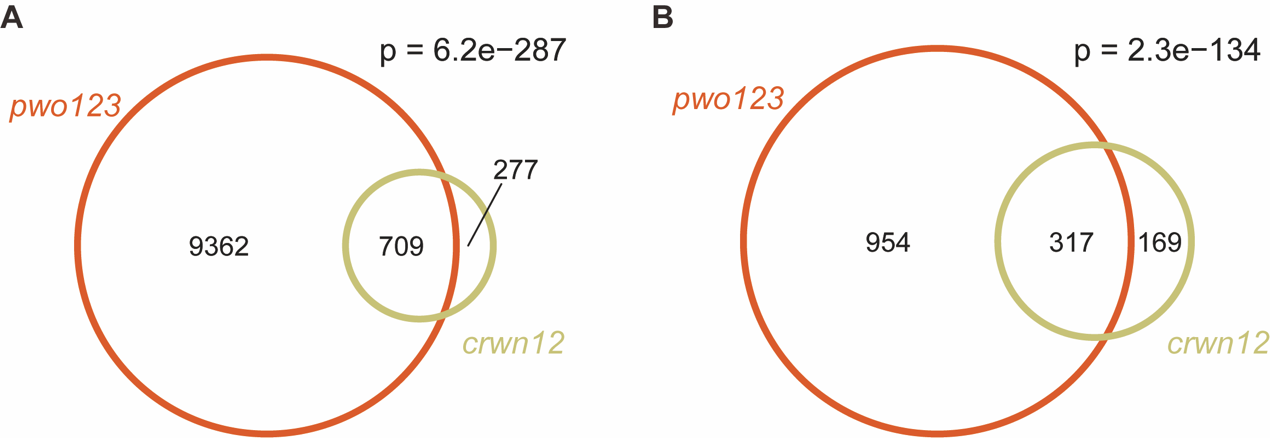


**Fig. S13 DEGs are significantly overlapped between *pwo123* and *crwn12* mutants*.* A** Venn diagrams showed the significant overlap of differentially expressed genes in *pwo123* and *crwn12* mutants. **B** Venn diagrams showed the significant overlap of differentially expressed genes within H3K27me3-CDs in *pwo123* and *crwn12* mutants.


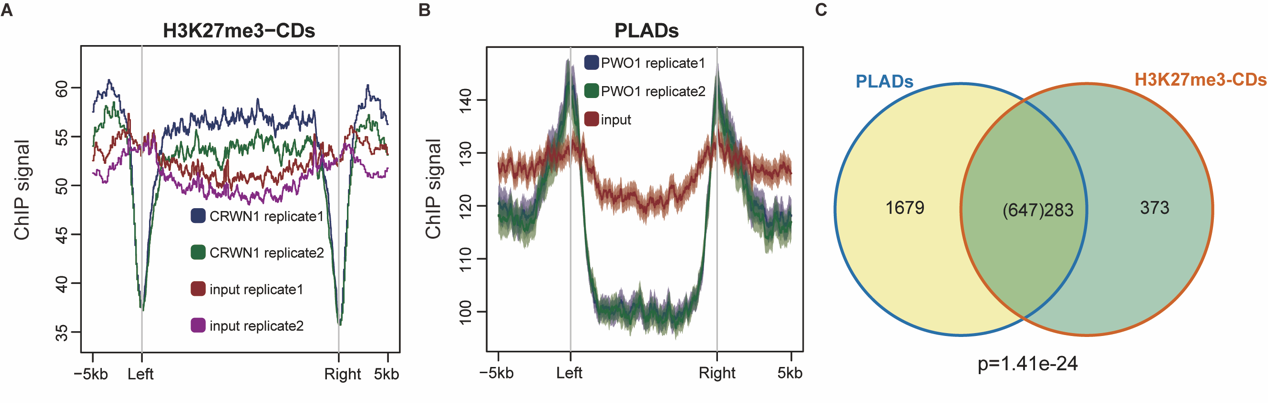


**Fig. S14 Overlap between H3K27me3-CDs and PLADs. A** The meta plot indicated that CRWN1 prefers to bind to inside of H3K27me3-CDs. **B** The meta plot showed PWO1 binds to the boundary of PLADs. **C** Venn diagram showed that 283 H3K27me3-CDs are significantly overlapped with 647 PLADs. Hypergeometric test was performed to calculate the p value.


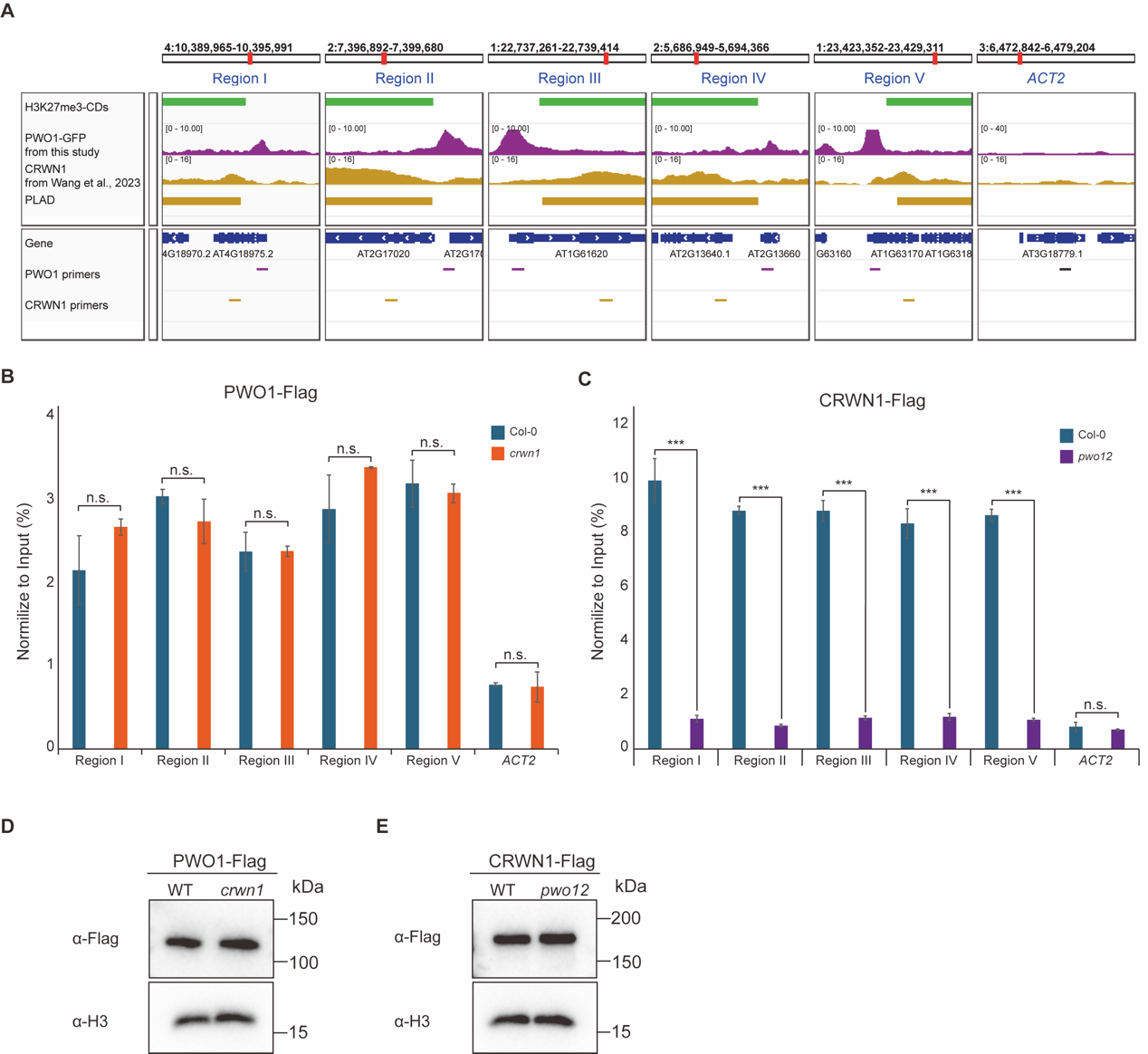


**Fig. S15 Relation of binding ability between PWO1 and CRWN1. A** Genome Browser views of PWO1-GFP or CRWN1 ChIP-seq signals at selected regions. The green or yellow blocks on the top or bottom indicated the H3K27me3-CDs or PLADs position. The purple or yellow lines below indicated the regions for ChIP-qPCR. **B** ChIP-qPCR result showed the PWO1-Flag binding in WT and *crwn1* background. **C** ChIP-qPCR result showed the CRWN1-Flag binding in WT and *pwo12* background. *ACT2* gene region was used as the negative control for both PWO1-Flag and CRWN1-Flag binding. **D-E** Western blot showed equal expression level of PWO1-Flag in WT or *crwn1* mutant background (D), or CRWN1-Flag in WT or *pwo12* mutant background (E). H3 was used as the loading control.
